# Computational identification of hepatitis C virus associated microRNA-mRNA regulatory modules in human livers

**DOI:** 10.1186/1471-2164-10-373

**Published:** 2009-08-11

**Authors:** Xinxia Peng, Yu Li, Kathie-Anne Walters, Elizabeth R Rosenzweig, Sharon L Lederer, Lauri D Aicher, Sean Proll, Michael G Katze

**Affiliations:** 1 Department of Microbiology, School of Medicine, University of Washington, Seattle, Washington, USA

## Abstract

**Background:**

Hepatitis C virus (HCV) is a major cause of chronic liver disease by infecting over 170 million people worldwide. Recent studies have shown that microRNAs (miRNAs), a class of small non-coding regulatory RNAs, are involved in the regulation of HCV infection, but their functions have not been systematically studied. We propose an integrative strategy for identifying the miRNA-mRNA regulatory modules that are associated with HCV infection. This strategy combines paired expression profiles of miRNAs and mRNAs and computational target predictions. A miRNA-mRNA regulatory module consists of a set of miRNAs and their targets, in which the miRNAs are predicted to coordinately regulate the level of the target mRNA.

**Results:**

We simultaneously profiled the expression of cellular miRNAs and mRNAs across 30 HCV positive or negative human liver biopsy samples using microarray technology. We constructed a miRNA-mRNA regulatory network, and using a graph theoretical approach, identified 38 miRNA-mRNA regulatory modules in the network that were associated with HCV infection. We evaluated the direct miRNA regulation of the mRNA levels of targets in regulatory modules using previously published miRNA transfection data. We analyzed the functional roles of individual modules at the systems level by integrating a large-scale protein interaction network. We found that various biological processes, including some HCV infection related canonical pathways, were regulated at the miRNA level during HCV infection.

**Conclusion:**

Our regulatory modules provide a framework for future experimental analyses. This report demonstrates the utility of our approach to obtain new insights into post-transcriptional gene regulation at the miRNA level in complex human diseases.

## Background

The hepatitis C virus (HCV) belongs to the *Flaviviridae *family and encodes a small enveloped, positive-stranded RNA genome of 9.6 kb [1]. HCV infects approximately 170 million people worldwide and infection often leads to serious chronic liver diseases including liver cirrhosis, liver failure and hepatocellular carcinoma [2,3]. This is attributed in part to the remarkable ability of the virus to establish persistent infections [4-7]. Current medical treatment for HCV involves the antiviral agent type 1 IFN combined with ribavirin, but only ~55% of treated patients have a sustained virologic response, i.e., the absence of detectable HCV RNA 24 weeks after cessation of therapy [8,9]. Clearly, there is a desperate need for new anti-HCV therapies.

miRNAs have been implicated as potential new targets for HCV therapy. miRNAs are a class of small non-coding RNA molecules of 20–22 nucleotides that regulate gene expression through translational repression and mRNA degradation [10]. miRNAs play key roles in regulating gene expression in a variety of organisms and are involved in crucial physiologic and pathologic processes [11-13]. In vitro studies have shown that the liver-specific miR-122 is required for HCV RNA replication [14], whereas IFN-induced miRNAs miR-196 and miR-448 directly target HCV genomic RNA for inhibition of viral replication [15]. However, a recent report showed that there was no correlation between miR-122 expression and viral load in human subjects with chronic HCV undergoing IFN therapy, cautioning the extrapolation of *in vitro *observations to human subjects with HCV infection [16]. As a new player among gene regulation mechanisms, the functions of miRNAs have not been fully clarified. The comprehensive delineation of the relationships between HCV infection and cellular miRNAs is crucial to better understand HCV pathogenesis and to develop novel therapeutic strategies. To the best of our knowledge, there has not been a systematic study using high-throughput technology to analyze the role of cellular miRNAs during HCV infection.

Precise identification of miRNA targets is essential for the functional characterization of individual miRNAs and a better understanding of complex human diseases. Multiple computational approaches have been developed to predict miRNA-target relationships using sequence information [17]. However, accurate prediction of physiologically active miRNA targets remains challenging. The observation that many miRNAs cause mRNA degradation of their targets provides opportunities to develop new approaches for target identification and validation using high-throughput expression profiling. Gene expression profiling data has been used to identify functional targets [18,19], and to improve target predictions [20,21]. When the expression of miRNAs and mRNAs are simultaneously profiled across different conditions, the miRNA and the mRNAs that it targets for degradation should exhibit an inverse expression relationship. A successful strategy for miRNA target identification has been reported using the inverse relationships between miRNAs and mRNAs inferred from the paired expression profiles across different conditions [22]. The utility of inverse correlation for miRNA target identification has been further demonstrated in more recent studies, such as surveys of miRNA and mRNA expression in human cell lines [23] and miRNA expression and protein abundance in rat kidneys [24]. This approach, unlike miRNA transfection, avoids artificial conditions needed to perturb gene expression in the systems of interest.

Identification of functional modules has greatly advanced our understanding of complex biological networks [25,26]. A single miRNA can regulate a large number of target genes in mammalian cells [27], and multiple miRNAs may regulate the same target [28]. To understand the many-to-many regulatory relationships in complex cellular systems, attempts have been made to predict miRNA regulatory modules. Yoon and De Micheli introduced the concept of such modules, or groups of miRNAs and target genes that are believed to participate cooperatively in post-transcriptional gene regulation [29]. Their modules are related only to miRNA-mRNA binding information at the sequence level. To improve module prediction, two different computational approaches have been proposed to integrate mRNA and miRNA expression profiles [30,31], using the same published expression dataset [32]. Both approaches introduced the measurement of coherent expression among miRNAs or mRNAs, but not between miRNAs and mRNAs. Recently, a graphical model approach was used to predict miRNA regulatory modules in *Arabidopsis *[33]. However, none of the above explicitly considers the inverse expression relationships for module prediction, which has been very effective in terms of uncovering functional miRNA-target relationships.

In this report, we present an integrative strategy for inferring HCV-associated miRNA-mRNA regulatory modules, by combining the inverse expression relationships between miRNAs and mRNAs and computational target predictions at the sequence level. We generated, for the first time, a systematic profiling of cellular miRNA expression during HCV infection in human livers. We inferred inverse expression relationships by simultaneous microarray profiling of host miRNA and mRNA expression across 30 human liver biopsies, including samples from HCV-infected and uninfected individuals. Using our integrative computational approach, we identified 38 miRNA-mRNA regulatory modules that were associated with HCV infection. We analyzed the functional roles of those modules at the systems level through the integration of a large protein interaction network. We show that the expression of multiple cellular miRNAs was altered and provide evidence that miRNAs are involved in a combinatorial and modular fashion in the regulation of host responses during HCV infection. Together, these results provide novel insights into regulatory mechanisms at the miRNA level during HCV infection, and our analytical approach shows the utility of an integrative strategy that may be applied to the study other complex human diseases for the identification of miRNA regulatory modules.

## Results

### Analysis strategy

**Figure 1:**
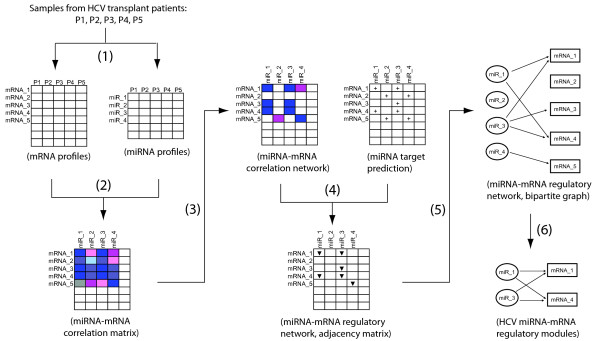
**Workflow of module identification**. 1) Profile expression of both miRNAs and mRNAs in the same set of samples using microarrays. 2) Calculate miRNA-mRNA correlation matrix based on the similarities in the expressions across samples. 3) Estimate false detection rates for a series of thresholds, and choose one based on the desired false detection rate to convert the correlation matrix into a binary miRNA-mRNA correlation network. 4) Construct a miRNA-mRNA regulatory network by combining the constructed miRNA-mRNA correlation network and the corresponding miRNA-target matrix. 5) Represent the regulatory network as a bipartite graph. 6) Enumerate all maximal bicliques as candidate regulatory modules, and post-process candidate modules, including the assessment of both the statistical significances, and differential expressions of target mRNAs between HCV+ and HCV-.

Our strategy for the identification of miRNA-mRNA regulatory modules integrated two independent, yet complementary types of information: inverse expression relationships and computational target predictions (Figure 1). The analysis of the gene expression data allowed identification of genes that were inversely co-expressed with a miRNA, whereas the target prediction was used to identify genes containing binding sites of a miRNA. We reasoned that the regulatory relationships identified using two sources of information are more likely to be physiologically functional than those identified using either one of them alone. Our approach consists of six major steps. The first three steps are for inferring the inverse expression relationships between miRNAs and mRNAs through the analysis of expression correlations.

In step 1, we collected the paired expression data through microarray profiling of the expression of both miRNAs and mRNAs across the same set of HCV liver biopsy samples. To focus on HCV infection, we restricted our analysis to those miRNAs differentially expressed between HCV positive (HCV+) and HCV negative (HCV-) samples. In step 2, we calculated the correlation for each miRNA-mRNA gene pair based on the gene expression patterns across different samples, and created one large miRNA-mRNA correlation matrix. In step 3, we first estimated the false detection rates associated with different thresholds for correlations. We then select an appropriate threshold based on the desired false detection rate, and converted the correlation matrix into a binary miRNA-mRNA correlation network. In parallel we obtained computational target predictions based on seed match, and created a miRNA-target matrix. In step 4, to integrate the inferred inverse expression relationships and target predictions, we combined the miRNA-mRNA correlation network and the corresponding miRNA-target matrix into a miRNA-mRNA regulatory network. In step 5, we represented the regulatory network as a bipartite graph. There were two disjoint sets of nodes in this graph, miRNA and mRNA genes. A direct connection was placed from a miRNA to a mRNA when: a) the expression of the miRNA was inversely correlated to that of the mRNA, and b) the mRNA was predicted to be the target of the miRNA.

Finally, in step 6 we first enumerated all maximal bicliques in the bipartitie graph and defined them as candidate regulatory modules. Next, for each candidate module we assessed the statistical significance, or the probability of finding it by chance, and the differential expression of target mRNAs between HCV+ and HCV- samples. The significant candidates, considered by both measurements, were identified as HCV associated miRNA-mRNA regulatory modules. We evaluated the direct miRNA regulation of its targets in a few identified modules using available miRNA transfection data [19]. We then performed functional analysis of miRNA target genes to study the biological roles that the miRNAs play during HCV infection. Each of these steps are detailed in the following sections.

### miRNA and mRNA expression in liver biopsy samples

In order to infer the inverse expression relationships between miRNAs and mRNAs during HCV infection, we generated a large paired miRNA and mRNA expression dataset. We collected 24 HCV positive (HCV+) and 12 HCV negative (HCV-) liver biopsy samples from 29 liver transplantation patients. After isolation of total RNA, the expression of 470 unique human miRNAs in all samples was profiled using microarrays. For 7 out of 29 patients, two samples were taken from the same individuals, but at different times (6–18 months apart) after transplantation. Since we did not observe significant correlations between samples from the same patients in terms of expression profiles, all 36 samples were treated as independent observations in the following analysis.

We first examined if the miRNAs showed differential expression between HCV+ and HCV- samples. We found multiple miRNAs were differentially expressed between HCV+ and HCV- samples, though large variations within each group of samples were observed (Additional file 1: Table S1). Among them, miR-122 was under expressed in the HCV+ samples (~1.8 fold, *P *= 1.5e-3,). As the most abundant microRNA specifically expressed in liver tissues, miR-122 has been shown to be involved in modulating HCV replication [14]. Also, miR-16 was up-regulated among HCV+ samples by ~2 fold (*P *= 9e-5). miR-16 is shown to induce cell cycle arrest [34], and perturbation of cell cycle progression may be a common scenario during HCV infection that impacts the severity of liver injury [35]. Furthermore, another cell-cycle associated miRNA, miR-320 [36], was down-regulated (~1.8 fold, *P *= 1.1e-2) in HCV+ samples.

To focus on HCV infection, and to simplify the downstream analysis, we limited our module analysis to those miRNAs differentially expressed between HCV+ and HCV- samples. Since it was unclear how much difference at the miRNA level could lead to detectable phenotypical differences, that is, the differential expression of target mRNAs, we used a relatively relaxed criteria, fold change > = 1.2 and *P *< 0.1, to select miRNAs differentially expressed between HCV+ and HCV- samples. In total, 54 miRNAs satisfied the criteria and were selected for future analysis (Additional file 1: Table S1).

In parallel, we proceeded to profile mRNA expression in 30 of 36 liver biopsy samples, for which sufficient materials were available, including 24 HCV+ and 6 HCV- samples. Many miRNAs derive from independent transcriptional units, but some miRNAs locate in the introns of protein-coding genes, so called miRNA host genes. Since it has been reported that miRNA host genes and intronic miRNAs tend to be co-expressed [37], we looked at the expression of miRNA host genes in these HCV related liver samples. Interestingly, we observed that the expression levels of some, but not the majority of miRNAs and their host genes were significantly correlated across these 30 liver biopsy samples. For example, the correlation between *miR-191 *and its host gene *DALRD3 *was 0.65 (Pearson coefficient), *P *= 9.8e-5. Similarly, the correlation between *miR-16 *and its host gene *SMC4 *was 0.55, *P *= 0.0016. This information may be useful for future studies on the regulation of miRNA expression.

### Construction of a miRNA-mRNA regulatory network

To systematically link the 54 miRNAs identified above to their potentially regulated target genes, we inferred the inverse expression relationships between those miRNAs and their target mRNAs using the generated expression data. For each identified miRNA, we calculated all pair wise correlations between the miRNA and every mRNA represented on the microarray based on their expression across all 30 samples. This resulted in a miRNA-mRNA correlation matrix, in which the rows are mRNA transcribing genes and the columns are miRNA genes. We then converted the correlation matrix into a binary matrix, a miRNA-mRNA correlation network, by applying a threshold on the correlations. Instead of choosing an arbitrary high threshold, we first estimated the false detection rates associated with a series of thresholds.

**Figure 2:**
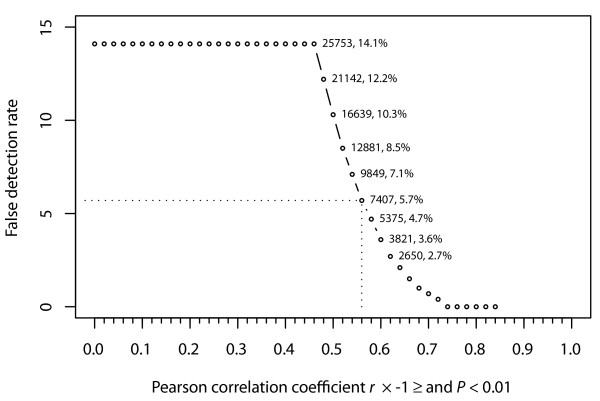
**Estimated false detection rates associated with different thresholds for miRNA-mRNA expression correlations**. The x-axis is a series of thresholds for the Pearson correlation coefficient. The requirement of *P *< 0.01 was applied for all thresholds at the same time. The y-axis has false detection rates for the corresponding thresholds. The dotted lines indicate that the estimated false detection rate is ~5.7% when the threshold is chosen as Pearson correlation coefficient *r *< = -0.56 and *P *< 0.01. For selected thresholds, the numbers aside show the total number of miRNA-mRNA pairs satisfying the corresponding threshold and the estimated false detection rate. The requirement of *P *< 0.01 alone has estimated false detection rate as ~14% in this dataset.

The false detection rate was defined as the percentage of miRNA-mRNA gene pairs out of the total number of selected pairs that would have the same or better correlations just by chance. To do so, we generated simulated datasets by randomly choosing the same number of arrays from the real expression data. Essentially, we randomized the correspondences among samples underlying the miRNA and mRNA expression data, though the expression data for each individual sample was the same. This simulation should enable us to better balance the trade-off between sensitivity (retaining more gene pairs) and specificity (maintaining high correlations within each gene pair) overall. We wanted to select a threshold which would have a relatively low false detection rate overall, but still leave us a large number of highly correlated miRNA-mRNA gene pairs. We chose the threshold value as Pearson correlation coefficient *r *≤ -0.56 and *P *< 0.01, for which we estimated that the overall false detection rate was ~5.7% (Figure 2). We repeated the simulation experiments independently three times, and obtained similar false detection rates. The resulting miRNA-mRNA correlation network had 7,407 connections between 54 miRNAs and 3,093 mRNAs. On average, one miRNA was highly correlated to ~137 mRNAs, and one mRNA to ~2.4 miRNAs (Additional file 1: Figure S1A).

Since the perfect match to the so-called seed region of 6–8 nt at the 5' end of miRNA has been shown to be a major determinant in the miRNA-target recognition process, we examined if those inversely correlated mRNAs were enriched with 6-mers matching the miRNA seed region in their 3' UTR. We found that 10 out of 54 miRNAs showed enrichment with seed 6-mers (Fisher exact test, *P*-value < 0.05). This suggests that inverse expression relationship was very informative in terms of identification of miRNA target genes, considering the complexities of the regulatory networks involved in the *in vivo *systems under study, and that only a portion of all potential targets functionally regulated at the mRNA level during HCV infection.

To investigate if miRNAs directly regulated the mRNA level of those genes exhibiting a highly inversely correlated expression profile, we superimposed miRNA target prediction information onto the miRNA-mRNA correlation network and created a regulatory network. In the regulatory network, a direct connection was placed from a miRNA to an mRNA when: a) the expression level of the miRNA was highly inversely correlated to that of the mRNA, and b) the mRNA was predicted to be the target of the miRNA. In this study, the miRNA target predictions were based on the seed matches only (See Materials and methods). On average, ~22% of correlated miRNA-mRNA pairs overlapped with predicted miRNA-mRNA pairs. The obtained regulatory network had 1,588 connections from 51 miRNAs to 1,077 mRNAs, 814 of which were also predicted miRNA-mRNA pairs by TargetScan. On average, one miRNA was connected to about 31 mRNAs, and one mRNA to 1.5 miRNAs (Additional file 1: Figure S1B).

To assess the broad HCV relevance of our regulatory network, we used Ingenuity Pathways Analysis (IPA) to perform pathway analysis of those 1,077 mRNAs. We were particularly interested in knowing if miRNAs targeted genes in pathways that have been shown to be associated with HCV infection. As shown in Table 1, our analysis suggests that cellular miRNAs may be involved in the regulation of multiple HCV-associated pathways. For example, the chemokine mediated immune response is pivotal to the clearance of virus during the acute HCV infection. Dysregulation of chemokine signaling pathways has been a common strategy exploited by a variety of viruses [38-40], and miRNAs can be convenient tools for viruses to implement that strategy [41]. Our results showed that miRNAs may be able to target as many as 16 genes in this particular pathway, including chemokine CXCL12 and its downstream gene PKC-β. CXCL12 has been shown to be regulated by HCV and able to induce HCV related fibrosis [42,43].

**Table 1:** Genes in HCV associated pathways identified as regulated by miRNAs during HCV infection

Canonical pathways (IPA)	Predicted target genes	Number of targets
Chemokine Signaling	SRC, CALM3, CALM2, MAPK6, PPP1CB, KRAS, LIMK2, PTK2, CAMK2D, RRAS2, MAPK14, CCL2, RHOA, CXCL12, CALM1, PRKCB	16

B Cell Receptor Signaling	MAP2K4, MAP3K14, CALM3, PIK3R1, CALM2, MAPK6, KRAS, INPPL1, PTEN, BCL2L1, CAMK2D, RRAS2, MAPK14, BCL10, PAG1, LYN, PIK3AP1, CALM1, PRKCB	19

PTEN Signaling	PIK3R1, MAPK6, INPPL1, KRAS, CCND1, PTEN, PTK2, BCL2L1, RRAS2, GHR, BMPR1A, CDKN1A, FASLG	13

IL-6 Signaling	MAP2K4, IL6ST, MAP3K14, MAPK6, MAP4K4, KRAS, IL1R1, STAT3, COL1A1, IL1F9, RRAS2, MAPK14, MAPKAPK2	13

ERK/MAPK Signaling	RAP1B, SRC, PIK3R1, MAPK6, PPP1CB, KRAS, STAT3, PPP2R5A, DUSP2, EIF4E, PLA2G4C, YWHAQ, PTK2, MAPKSP1, RRAS2, ELF3, PRKCB, PRKAR1A	18

JAK/Stat Signaling	RRAS2, PIK3R1, CDKN1A, SOCS6, MAPK6, KRAS, STAT3	7

As another example, 13 genes in the tumor suppressor phosphatase and tensin homolog (PTEN) signalling pathway were predicted to be targeted by miRNAs during HCV infection. PTEN is frequently mutated in a large number of cancers, and PTEN deletion mice demonstrate chronic liver injury and steatosis prior to the development of primary liver carcinomas[44], events that also coincide with the progression of HCV-associated human liver diseases. As a miRNA target [45-47], PTEN was down-regulated in the HCV infected livers. The ERK/MAPK singling pathway plays a central role in intracellular signalling network and is associated with a number of key biological functions [48]. Multiple lines of evidence indicate the important roles of the ERK/MAPK pathway in HCV replication and viral gene expression [49,50]. Our results identified 18 genes in the ERK/MAPK pathway, including some key members such as Ras (KRAS), 14-3-3 (YWHAQ), ERK3 (MAPK6) and STAT3, which may be targeted by miRNAs during HCV infection. Together, this result clearly indicates that genes in our regulatory network are closely related to HCV infection in general. Also our results suggest that cellular miRNAs have a broad impact on the regulation of host responses during HCV infection, and provide novel insights into the regulation of these HCV-associated pathways at the miRNA level.

### Identification of miRNA-mRNA regulatory modules

To uncover the many-to-many regulatory relationships between miRNAs and their functional target genes, and to generate testable hypotheses for future experiments, we attempted to predict miRNA-mRNA regulatory modules in the constructed regulatory network. A miRNA-mRNA regulatory module consists of a set of miRNAs and a set of their targets, in which the miRNAs co-ordinately regulate their targets. To ensure that every miRNA was connected to every mRNA in the same module, we modelled each regulatory module as a biclique in the regulatory network, which essentially was a bipartite graph. We focused on maximal bicliques to avoid redundancy. In total, we identified 284 maximal bicliques as our candidate regulatory modules. The maximum number of miRNAs and the maximum number targets in a candidate module were 7 and 191 respectively, but the majority of modules had less than 3 miRNAs and 5 mRNA targets (Additional file 1: Figure S2). Since miRNAs tend to regulate large number of targets, we chose to further analyze only those candidate modules with relatively more mRNAs, i.e., modules with 10 or more mRNAs In total 47 out of 284 candidates were kept, which covered ~90% of the targets in the regulatory network (Additional file 1: Figure S3).

For each of these 47 predicted regulatory modules, we estimated the *P *value, or the probability of finding it by chance, using permutation based tests. We developed two different metrics, the joint probability based P_score and the counting based P_Nt (see Materials and methods for details). Since P_score takes into account the strengths of individual correlations and the different abundances of predicted miRNA targets, we expected it to be more accurate. But it is computationally intensive, as it searches for one set of miRNAs and mRNAs with the maximum score among all possible combinations of miRNAs and mRNAs of the same size of the candidate modules. This might not be feasible for candidate modules of even slightly larger sizes. However, P_Nt counts the maximum number of targets that would be predicted to be regulated by the same set of miRNAs as the candidate module using random datasets. Therefore it is much more computationally efficient, but possibly less accurate.

At the significance level of *P *value < 0.05, 4 out 47 candidate modules were considered as insignificant by P_score and 5 by P_Nt, based on the analysis of 100 random datasets. Interestingly, all four candidates considered insignificant by P_score were also considered insignificant by P_Nt. This agreement indicates that these four candidates could indeed happen by chance. More importantly, it suggests that we might be able to use the metric P_Nt as an approximation for the metric P_score in the future, though more thorough comparisons are needed. However, in this study we deemed a candidate module as statistically significant only if it was considered as significant by both metrics. This left us with 42 modules. We repeated the simulation experiments independently three times with similar results.

In parallel, we wanted to enrich those modules in which targets were significantly differentially expressed between HCV+ and HCV- samples. Our hypothesis was that genes showing larger differences between HCV+ and HCV- samples were more likely to be related to HCV infection. For each of 47 candidate modules, we also assessed the differential expression of targets between HCV+ and HCV- samples. Instead of testing one gene at a time, we treated all targets within a module as one group and evaluated the overall expression differences (See Materials and methods). This was expected to be more robust and sensitive. Out of those 47 candidate modules with 10 or more targets, 42 had an adjusted *P *value of less than 0.05 and were considered as HCV associated. We repeated the simulation experiments for the calculation of adjusted *P *value independently three times, and obtained same results.

By intersecting two lists from assessments above, we identified 38 miRNA-mRNA regulatory modules that were both statistically significant (module *P *value) and HCV associated (differential expression). Table 2 shows the summary of these identified modules (Additional file 2 has details, including gene list, miRNA-target correlation and target prediction). Since our target predications were based on the 6-mer seed match alone, we decided to examine if targets in our identified modules were also targets predicted by other computational approaches. Noticeably, we found that on average ~52% of targets in our predicted modules were also predicted as targets of the corresponding miRNAs by TargetScan (Table 2), one of the better computational predictions as suggested by [51]. When we compared our predictions with the conserved targets from TargetScan predictions, we found that on average ~11.4% of the targets in our identified modules were also conserved (i.e., having at least one conserved site as predicted by TargetScan, Table 2). This agreement suggests that our integrative strategy enriched for true functional miRNA-target relationships. A strict requirement on evolutionary conservation might significantly increase false negatives.

**Table 2:** Summary of identified miRNA-mRNA regulatory modules

						mRNA expression in HCV+ vs. HCV-^b^			Module p-value^c^		
Module identifier	No. of mRNA	No. of miRNA	miRNA	miRNA gene family	% of overlapped targets^a^	log2 ratio	p-value	adj. p-value	P_score	P_Nt	Selected GO terms (BP)^d^

**miRNA down-regulated, and mRNA up-regulated in HCV+ vs. HCV-**											

7	48	1	hsa-miR-320	mir-320	56.2 (12.5)	0.6	4.8E-09	0.001	1.1E-25	0	cell cycle checkpoint

8	34	1	hsa-miR-92	mir-25	44.1 (20.6)	0.6	5.6E-10	0	1.3E-33	0	actin cytoskeleton organization and biogenesis

10	77	1	hsa-miR-296	mir-296	62.3 (2.6)	0.8	4.3E-15	0	5.5E-37	0	T cell receptor signaling pathway

29	37	1	hsa-miR-193b	mir-193	43.2 (2.7)	0.6	3.9E-08	0	3.3E-33	0	regulation of apoptosis

30	39	1	hsa-miR-181b	mir-181	59 (5.1)	1.0	5.8E-24	0	2.8E-22	0.03	cell cycle checkpoint

37	46	1	hsa-miR-422b	mir-378	39.1 (2.2)	0.5	6.4E-07	0	8.1E-26	0	B cell differentiation

39	74	1	hsa-miR-122a	mir-122	51.4 (1.4)	0.5	1.7E-05	0.011	1.4E-44	0.01	DNA repair

50	11	2	hsa-miR-122a, hsa-miR-320	mir-122, mir-320	54.5 (0)	0.8	4.9E-13	0	1.3E-56	0	MAPKKK cascade

87	11	2	hsa-miR-193b, hsa-miR-320	mir-193, mir-320	31.8 (0)	0.5	2.1E-06	0.001	2.8E-53	0	cell cycle checkpoint

**miRNA up-regulated, and mRNA down-regulated in HCV+ vs. HCV-**											

1	52	1	hsa-miR-130a	mir-130	46.2 (19.2)	-0.5	2.3E-05	0.016	6.5E-15	0.02	regulation of Rho protein signal transduction

2	66	1	hsa-miR-26b	mir-26	60.6 (13.6)	-0.7	3.6E-08	0	7.9E-32	0	actin cytoskeleton organization and biogenesis

4	191	1	hsa-miR-16	mir-15	54.5 (18.3)	-0.8	2.5E-09	0	2.6E-46	0	insulin receptor signaling pathway

13	51	1	hsa-miR-21	mir-21	49 (5.9)	-0.6	7.6E-07	0.002	3.8E-33	0	inactivation of MAPK activity

15	170	1	hsa-miR-26a	mir-26	62.4 (17.1)	-0.6	6.4E-07	0.002	4.8E-50	0	insulin receptor signaling pathway

16	22	1	hsa-miR-155	mir-155	54.5 (4.5)	-0.6	7.7E-10	0	4.7E-17	0.02	B cell differentiation

18	59	2	hsa-miR-26a, hsa-miR-26b	mir-26	62.7 (15.3)	-0.6	3.2E-07	0.001	7.8E-85	0	innate immune response

27	86	1	hsa-miR-215	mir-192	43 (1.2)	-0.7	1.8E-07	0.002	8.2E-32	0	insulin receptor signaling pathway

28	21	2	hsa-miR-16, hsa-miR-215	mir-15, mir-192	38.1 (9.5)	-0.7	7.0E-09	0	2.2E-51	0	apoptotic program

36	37	1	hsa-miR-324-3p	mir-324	45.9 (0)	-0.6	2.9E-08	0	2.6E-07	0	cAMP-mediated signaling

43	88	1	hsa-miR-202	mir-202	43.2 (13.6)	-0.6	2.3E-04	0.036	5.6E-52	0	apoptotic program

52	82	1	hsa-miR-509	mir-509	50 (1.2)	-0.6	1.6E-11	0	4.8E-60	0	transforming growth factor beta receptor signaling pathway

55	48	1	hsa-miR-424	mir-322	60.4 (20.8)	-0.7	1.2E-09	0	1.3E-13	0.02	nuclear import

56	30	2	hsa-miR-16, hsa-miR-424	mir-15, mir-322	53.3 (23.3)	-0.7	1.5E-09	0	1.1E-58	0	transforming growth factor beta receptor signaling pathway

57	12	1	hsa-miR-191	mir-191	66.7 (0)	-0.6	4.0E-07	0.002	7.8E-51	0	positive regulation of peptidyl-serine phosphorylation

58	15	2	hsa-miR-26a, hsa-miR-424	mir-26, mir-322	66.7 (23.3)	-0.7	2.1E-10	0	1.4E-44	0	transforming growth factor beta receptor signaling pathway

59	19	2	hsa-miR-16, hsa-miR-26a	mir-15, mir-26	60.5 (26.3)	-0.7	4.4E-11	0	2.4E-62	0	transforming growth factor beta receptor signaling pathway

60	11	3	hsa-miR-16, hsa-miR-26a, hsa-miR-424	mir-15, mir-26, mir-322	54.5 (33.3)	-0.7	5.3E-12	0	2.5E-67	0	transforming growth factor beta receptor signaling pathway

63	28	1	hsa-miR-199a*	mir-199	0 (0)	-0.8	5.0E-07	0.002	2.1E-04	0.03	negative regulation of MAP kinase activity

73	12	2	hsa-miR-16, hsa-miR-509	mir-15, mir-509	66.7 (25)	-0.6	2.0E-09	0	4.2E-55	0	negative regulation of translational initiation

76	28	2	hsa-miR-215, hsa-miR-26a	mir-192, mir-26	57.1 (12.5)	-0.6	6.7E-07	0	1.0E-66	0	transforming growth factor beta receptor signaling pathway

78	23	2	hsa-miR-130a, hsa-miR-16	mir-130, mir-15	47.8 (10.9)	-0.4	1.3E-07	0.001	9.6E-63	0	RNA-mediated gene silencing

79	35	1	hsa-miR-146b	mir-146	68.6 (2.9)	-0.3	5.5E-08	0	4.7E-14	0	complement activation, alternative pathway

81	23	1	hsa-miR-15b	mir-15	30.4 (8.7)	-0.3	2.3E-04	0.019	6.7E-05	0.03	telomere maintenance via telomerase

94	23	2	hsa-miR-202, hsa-miR-509	mir-202, mir-509	56.5 (6.5)	-0.4	4.3E-06	0.005	2.0E-89	0	transforming growth factor beta receptor signaling pathway

96	15	2	hsa-miR-21, hsa-miR-26a	mir-21, mir-26	53.3 (23.3)	-0.5	1.7E-08	0.001	1.6E-51	0	MAPKKK cascade

101	11	3	hsa-miR-21, hsa-miR-26a, hsa-miR-26b	mir-21, mir-26	54.5 (21.2)	-0.4	1.0E-05	0.005	8.8E-72	0	innate immune response

102	10	3	hsa-miR-215, hsa-miR-26a, hsa-miR-26b	mir-192, mir-26	53.3 (20)	-0.6	5.9E-07	0.003	1.4E-75	0	endothelial cell migration

170	10	2	hsa-miR-202, hsa-miR-324-3p	mir-202, mir-324	50 (10)	-0.5	1.0E-07	0.001	1.1E-32	0	response to dsRNA

^a^The overlap (%) is with the TargetScan prediction. The number in parenthesis is the overlap (%) with TargetScan conserved predicted targets, i.e. those targets with a conserved site as predicted by TargetScan.

^b^Differential expression of target mRNAs in HCV+ vs. HCV- was assessed together as one group for each module. adj. p-value: adjusted p-value, estimated based on 1000 permutations.

^c^Module p-value was estimated based on 100 simulations. P_score: the scoring method. P_Nt: the counting method.

^d^One representative GO term was manually selected for each module. See text for details.

### Evaluation of miRNA target regulation by use of in vitro miRNA transfection data

Since the connections between miRNAs and target mRNAs in our predicted modules were inferred based on inverse expression relationships and computational target predictions, it was unknown if those miRNAs could actually regulate their target genes directly. We therefore assessed the relationships using independent miRNA transfection studies. Linsley et al reported that each of 24 selected miRNAs was transfected into cell lines *in vitro*, and genome-wide mRNA expression was profiled both before and after each transfection [19]. The down-regulation of target mRNA level upon the miRNA transfection would indicate a direct regulation by the transfected miRNA.

**Figure 3:**
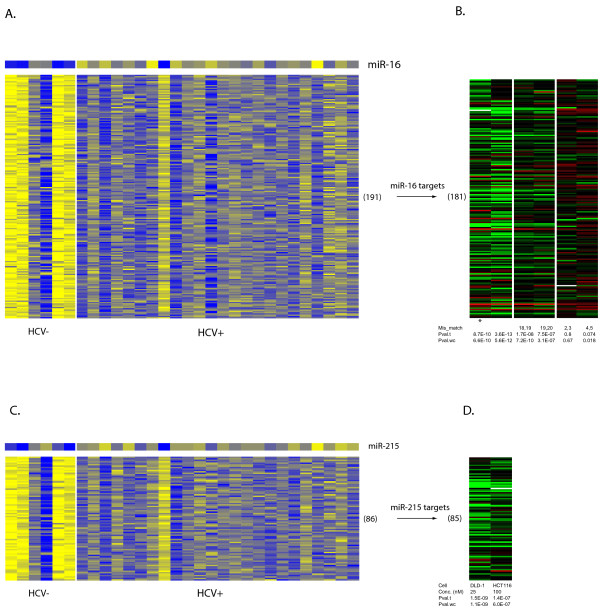
**Evaluation of direct miRNA regulation of target mRNAs using *in vitro *transfection data**. **A**. Heatmap showing inverse expression patterns between miR-16 and its 191 targets (module # 4 in Table 2). Top row shows miR-16 expressions across 30 liver biopsy samples, rows below for its targets. Columns represent samples, identical for both miRNAs and mRNAs. Expressions converted into z-scores. Entry in yellow (blue) indicates expression level of a gene is higher (lower) than mean across all samples. **B**. Heatmap showing down-regulation of miR-16 targets after transfection into cultured cells. Rows are targets and columns are experiments. The measurements for 181 of 191 targets available, and shown as log2 ratios of 24 h after transfection to mock. Transfections were done with 100 nm miRNAs using HCT116 Dicer-/- cells, the first with 25 nm miRNAs using DLD-1 Dicer -/- cells. In table at the bottom, row 'Mis_match' shows if miR-16 with mismatches used, indicated by locations. 18,19 represents mismatches at positions 18 and 19 of miR-16 mature sequences counted from 5'. 18,19 and 19,20 (2,3 and 4,5) outside (within) seed region. One-sample t-tests (Pval.t for p-values) and non-parametric Wilcoxon tests (Pval.wc) used to assess if overall target expressions shifted after transfection. **C**. Heatmap showing inverse expression patterns between miR-215 and its 86 targets (module #27 in Table 2), similarly as in (**A**). **D**. Heatmap showing down-regulation of miR-215 targets after transfection, similarly as in (**B**). The measurements for 85 of 86 targets were available. Table at the bottom shows cell types (Cell) and concentrations ('Conc.'). The transfection data was downloaded from GEO: GSM156550, GSM156546, GSM156565, GSM156566, GSM156563, GSM156564 for data shown in (**B**) from left to right, and GSM156552, GSM156548 in (**D**). See Linsley et al, 2007 for details.

Out of 24 miRNAs studied, three of them, miR-16, miR-215 and miR-15b, happened to be in at least one of our predicted modules (Table 2). We looked at the targets in each of those 3 modules with a single regulatory miRNA. We found that targets in 2 out of 3 predicted modules were significantly down-regulated by the corresponding miRNAs, miR-16 and miR-215 (Figure 3). Remarkably, the mismatches within the seed region (at positions 2 and 3, and 4 and 5) reversed the down-regulation of those predicted targets, but not mismatches outside the seed region (at positions 18 and 19, and 19 and 20) (Figure 3B). Obviously the *in vitro *system differed significantly from real human livers. However, the result strongly suggests that miRNAs were able to regulate many, if not all, of our predicted targets directly. More importantly, it clearly indicates that the direct regulation of mRNAs by miRNAs may be predicted with a high success rate using our integrative strategy.

### Integrative functional analysis

As shown in Table 2, some regulatory modules had more than one miRNA, indicating that those miRNAs co-ordinately regulated the same targets. At the same time, some miRNAs appeared in multiple regulatory modules, suggesting individual modules were interconnected. To better understand the biological functions of identified regulatory modules, we examined them at the systems level by looking for interaction partners for predicted miRNA targets in a large protein-interaction network. For each predicted regulatory module, we performed functional analysis of its targets plus direct interaction partners of those targets in the protein interaction network. Specifically, we analyzed the enrichment of Gene Ontology (GO) terms in the biological process category or specific KEGG pathways. This analysis may also be applied after combining several identified modules.

**Figure 4:**
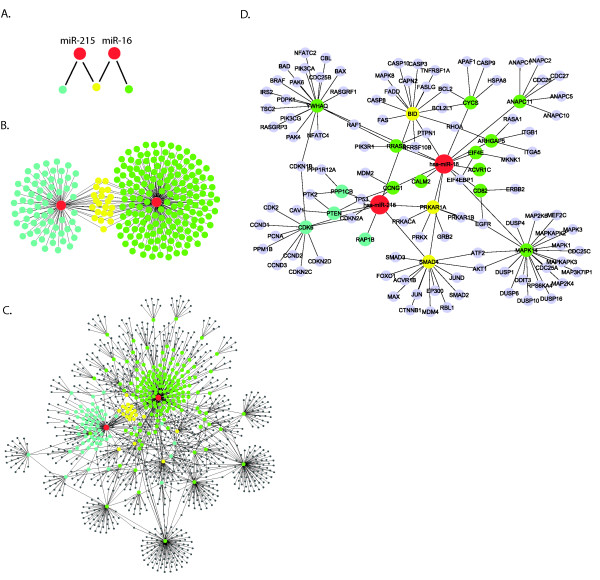
**An example of combined analysis of three identified regulatory modules**. Schema showing the relationships among module 4, 27, and 28 in Table 2. miRNAs are in red, and mRNA targets are in turquoise (miR-215), or in yellow (miR-215 and miR-16), or in green (miR-16). **B**. Combined overview of three modules, including miR-215, miR-16, and 86 miR-215 targets and 191 miR-16 targets (21 targets in common). **C**. An overview of the network expanded from **B**, by adding protein interaction partners of targets (black dots). Lines indicate protein interaction or miRNA-mRNA regulation. **D**. A sub-network in (**C**). Shown are genes in significantly enriched (*p*-value < 0.01), and HCV associated (manually selected based on literatures) KEGG pathways, including Cell cycle, Apoptosis, P53, MAPK, Insulin, Focal adhesion, Jak-Stat, TGF-beta, Toll-like. Each pathway had at least 10 member genes, including 2 or more predicted targets in (**C**).

To illustrate the complexity of combinatorial regulations at the miRNA level, we show in Figure 4 a combined network of three identified modules involving two miRNAs, miR-16 and-215, that were up-regulated in HCV+ samples. It should be noted that these two miRNAs and their targets may also be involved in other modules with different miRNAs and mRNAs (Table 2). Both miRNAs targeted large number of genes, 191 for miR-16 and 86 for miR-215, and 21 genes were targeted by both (Figure 4A and 4B, module # 4, 27 and 28 in Table 2). In the protein interaction network, miR-16 and miR-215 targets interact with many other proteins, including targets of the same miRNA and another miRNA (Figure 4C). From this network, we extracted a sub-network which included genes in several enriched KEGG pathways that are known to be associated with HCV infection (Figure 4D).

This sub-network suggests that HCV may be able to repress the expressions of several key genes in a number of HCV pathways via miR-16, miR-215, or both. For example, miR-16 targets MAPK14 (p38 MAPK) and BID (also a miR-215 target), which are two key regulators in apoptosis-associated pathways [52,53]. The induction of apoptosis among virus-infected cells is an effective host mechanism against virus infection. MAPK14 could be activated by inflammatory cytokines induced by virus infections and subsequently activate the downstream effectors that participate in the induction of IFN-dependent gene transcription [54]. This process may lead to apoptosis of virus-infected cells. Activated by the signalling from the proinflammatory chemokines, BID in turn activates mitochondria-mediated apoptosis. Intriguingly, in a chimeric mouse-human model the activation of BID results in a considerable decline of HCV RNA in serum, which suggests the antiviral property of BID [55]. By repressing the expression of MAPK14 and BID via miRNAs, HCV may be able to hinder apoptosis before ensuring successful establishment of virus replication at an early stage of infection.

We also observed miRNA targeting of Smad4, CD82 and PTEN (Figure 4D), which function as suppressors in tumorigenesis and metastasis. Smad4 is a central component of the TGF-β pathway, and degradation of Smad4 in tumors could specifically inhibit the tumor suppressor effect of TGF-β [56]. Similarly, down-regulation of the metastasis suppressor CD82 is correlated with cancer progression and poor survival in patients [57]. Furthermore, elevating the degradation of CD82 promotes tumor metastasis in mice [58]. PTEN mutations are observed in a wide range of cancers. More importantly, liver-specific loss of PTEN leads to rapid development of HCC in mice [59]. Therefore, by down-regulating the expression of these tumor suppressor genes including Smad4, CD82 and PTEN through cellular miRNAs, a persistent HCV infection could eventually lead to tumor development and metastasis in liver.

To obtain an overview of the functional impact of all modules on HCV infection, we attempted to annotate each module with a representative biological function. To do this, we looked for GO terms in the biological process category enriched in each individual module (target mRNAs plus interaction partners), as GO annotations have much better coverage of the whole genome than KEGG pathways and Ingenuity canonical pathways. We annotated each module by manually selecting one of the significantly enriched (*P *< 0.01) GO terms (Table 2). The selection of the GO term was judged by its association with HCV infection as indicated in previous publications, and the ratio of the number of genes in the module versus the total number of genes annotated to that particular GO term. Our selected biological functions included cell cycle checkpoint, innate immune response and negative regulation of translational initiation, which all have been extensively reported to be related to HCV infection. Overall, our findings suggest that our identified regulatory modules covered various biological processes that may be perturbed during HCV infection through cellular miRNA regulation, and that the miRNA regulation may occur in a combinatorial fashion as suggested by the analysis of regulatory modules. It would also be interesting in the future to investigate if these regulatory modules preferentially target similar components in large signaling networks as reported [13,60].

## Discussion

Twenty years since the identification of HCV, virologists and clinicians are still struggling to understand the underlying cause of HCV-related liver diseases. The recent discovery of the roles of miRNAs in many human diseases suggests that studies exploring the relationships between HCV and host miRNAs may provide new insights into HCV pathogenesis. In this study, we devised an integrative strategy to systematically investigate cellular miRNAs and their roles in gene regulation during HCV infection. Our computational approach integrates two independent, but complementary types of information: the inverse expression relationships inferred from the paired expression profiles of miRNAs and mRNAs, and the computational target prediction based on sequence information.

### Profiling miRNA and mRNA expression and inferring inverse expression relationships

The systematic identification of inverse expression relationships relies on the simultaneous transcriptional profiling of miRNAs and mRNAs across the same of set of samples. Although this can be laborious and costly, and the robustness of the functional miRNA-target relationships inferred from expression data depends upon the number of available samples, it may be beneficial for many studies. miRNA profiling provides another layer of valuable information about complex cellular systems. For example, it has been shown that miRNA signatures, but not mRNA signatures, are able to discern poorly differentiated tumors successfully [32]. miRNA profiling will be more accessible with the rapid development of high-throughput technologies such as miRNA microarrays and next-generation sequencing. Although it would be also informative to measure protein abundance changes [24,51,61], mRNA profiling is more convenient at this time, and studies profiling both miRNAs and mRNAs are emerging [32,62]. It is possible to use a miRNA host gene as a proxy for the expression of the intronic miRNA [63], but its application might be limited considering that many miRNAs do not have host genes. Also miRNAs and their host genes may be regulated differently even they are from the same primary transcripts.

For a number of reasons, inferring inverse expression relationships may be particularly attractive for studies using clinical samples. First, it is not possible to perturb gene expression in humans as required by some techniques like *in vitro *miRNA transfections. Second, relatively large individual variations are expected for studies using clinical samples in general in comparison to using model experimental systems. Whereas large variations could be a significant challenge for conventional approaches, such as the identification of differentially expressed genes, the analysis of correlations actually takes advantage of those variations. It infers functional relationships by examining correlated changes in expression across different samples and conditions.

### Computational prediction of miRNA targets in conjunction with inverse expression relationships

Precise target prediction remains challenging, especially when the predictions are based on sequence information alone. On the basis of several considerations, we predicted miRNA targets based on the seed matches alone. First, the fact that the existing computational target predictions differ strongly [64] makes a rational selection of one list of predictions over another extremely challenging. It remains difficult to assess accurately their performance, in part because of the limited number of experimentally validated targets and the diversities of datasets being used for the evaluation [17,65]. The second consideration is coverage. We observed that most computational techniques for miRNA target predictions start with the identification of potential targets based on the seed matching between miRNAs and mRNAs [17]. Without additional functional data, multiple rounds of filtering may be used to achieve a higher degree of specificity (fewer false-positives) as compared to sensitivity (fewer false negatives) based on various features like binding affinity and evolutionary conservation [17]. Although those features are useful for the enrichment of true miRNA targets in general, the criteria for filtering may have nothing to do with HCV infection in human livers, the primary biological interest here. Therefore, the filtering could eliminate many biologically important targets. Finally, we reasoned that after filtering based on significant inverse expression relationships, many of the remaining miRNA-mRNA pairs would already be functional miRNA-target pairs. For example, we were able to filter ~20,000 genes represented on the array down to ~3,000 genes in our constructed correlation network, based on the inverse expression relationships alone. Each miRNA was highly correlated to ~130 mRNAs on average, which was in the typical reported range of 100–200 targets per miRNA [27,28].

Considering that the seed match between miRNA and target is one of the most accurate predictors of miRNA targeting, and that most computational techniques for miRNA target predictions mainly rely on this information, we predicted targets based on this information alone for use in this study. By combining paired gene expression profiling, we expected to obtain reliable miRNA-target relationships physiologically active in our studied systems, while with a much better coverage. Later, when we compared our results with the target list from TargetScan, one of better available computational predictions, we found that more than half of the targets in our predicted modules were also considered as targets by their predictions, and many of them were also evolutionarily conserved. It was also encouraging that targets in two out of three predicted modules were significantly down-regulated in independent miRNA transfection experiments. These promising results strongly support our initial decision, but direct experimental validations will be critical for future studies. In principle, our strategy can directly take any single one or some combination of existing predicted target lists easily, but it would be informative to know what kind of features and filtering would be more effective when combined with inverse expression relationships.

### Gene expression coherence within miRNA-mRNA regulatory modules

Within a regulatory module, the expression of member genes is expected to be tightly correlated. Previous approaches for predictions of miRNA regulatory modules considered as a measurement the expression coherence; that is, that the expression of member miRNAs (and their targets) would be highly correlated within a module. This was not explicitly taken into consideration in our approach. However, we found that in all of our identified modules, the expression of miRNAs (Pearson correlation coefficient 0.86 on average for all modules) and mRNAs (0.6) were highly correlated with each other.

At least three factors may have contributed to the expression coherence observed in our predictions. First, expression coherence could be a by-product of inferring the inverse expression relationships. By requiring that all mRNAs must be highly correlated to each of the same set of miRNAs, we automatically enriched for a set of mutually highly correlated mRNAs. The same was true for miRNAs. Second, those miRNAs selected for module analysis were all differentially expressed between HCV+ and HCV- samples, therefore the expression of those miRNAs tended to be similar overall. Finally, we used a statistical model to select only those modules where all targets within the module were differentially expressed between two those groups of samples. Obviously, modules with targets of similar expression profiles would have better *P *values. Not only does our approach ensure coherent expression within each predicted module, but also it prevents the predictions from drifting away from our primary interest; that is, differences related to the presence or absence of HCV infection.

### Using data from an *in vitro *system to validate direct regulations

In this study, we used data from an *in vitro *miRNA transfection study to evaluate the miRNA-target regulatory relationships that we derived from data collected from human liver biopsies. Although the experimental systems are not equivalent, the use of the *in vitro *data provided a valuable and convenient approach to quickly assess the predicted miRNA regulation in general. Until recently, knowledge of HCV infection has been limited by the lack of a cell culture model supporting full HCV replication and by the absence of convenient animal models [66]. Complete replication of HCV has been achieved *in vitro *recently, though the chimpanzee remains the only animal model for HCV infection. Perturbing more relevant *in vitro *systems like HCV JFH-1 infection system in Huh7.5 cells, using techniques such as miRNA transfection, will facilitate the validation of predictions in the future.

## Conclusion

We have developed an integrative approach to identify HCV infection-associated miRNA-mRNA regulatory modules by combining paired miRNA and mRNA expression data and computational target prediction. Using this technique, our comprehensive analysis of the first systematic expression profiling of cellular miRNAs and mRNAs in HCV infected human livers indicates that miRNAs have wide impact on gene regulation during HCV infection. This regulation occurs in a combinatorial and modular fashion. These results provide new insights into the regulation of host responses and HCV pathogenesis. Our approach may be applied to the study of miRNA regulation in other complex human diseases.

## Methods

### Profiling of miRNA and mRNA expression

#### Liver biopsy samples

Twenty four HCV positive (HCV+) and 12 HCV negative (HCV-) tissue samples from percutaneous core needle biopsy specimens from liver transplantation patients were obtained according to clinical protocols at the University of Washington Medical Center. Following informed consent at the time of acquisition, all research samples were collected, flash frozen, and stored at -80°C. In total, samples from 29 patients were included in this study. The study was approved by the Institutional Review Board for Human Subject Review. For 30 of 36 liver biopsy samples, we obtained sufficient materials to profile both miRNA and mRNA expressions, which were used for inferring miRNA-mRNA functional regulatory relationships. Biopsy samples were from 19 HCV+ and 6 HCV- patients following liver transplantation. For this group of patients, liver recipient ages ranged from 24 to 72, with an average of 55 years, while ages of the liver donors ranged from 13 to 58, averaging 32 years. HCV negative patients were transplanted on account of autoimmune hepatitis, alcohol-induced cirrhosis or primary biliary cirrhosis. All other patients were transplanted on account of HCV-induced cirrhosis, gave evidence of recurrent HCV infection after transplant, and had intrahepatic HCV titers averaging 54,800 copies per 100 ng RNA at the time of biopsy. For 4 of these 30 liver biopsy samples, the transplantation patients had developed fibrosis by the time those biopsies were taken.

#### Sample preparation and RNA extraction

Tissue samples from liver biopsies were homogenized in Trizol reagent (Invitrogen, CA). The total RNA was isolated according to the manufacturer's protocol (Invitrogen). The integrity of miRNA and total RNA was confirmed by Bioanalyzer (Agilent small RNA kit and Nano kit). Prior to being included in the microarray analyses, the samples were tested for HCV RNA by qRT-PCR.

#### miRNA microarray analysis

miRNA gene expression profiling was carried out using Agilent human miRNA microarray Version 1 (Agilent Technologies, Palo Alto, CA), similarly as in [67]. Briefly, the miRNA microarray was designed based on the Sanger miRBase release 9.1, and contain 20–40 features targeting each of 470 human miRNAs (Agilent design ID 016436). Total RNA (100 ng) was used for making miRNA probes according to the Agilent protocol. Probes were hybridized at 55°C for 22 hours. Then the slides were washed by Gene expression wash buffer 1 at 16°C for 5 minutes and by Gene expression wash buffer 2 at 37°C for 5 minutes.

After hybridization and washing, the slides were scanned using an Agilent slide scanner. Microarray results were extracted using Agilent Feature Extraction software. We processed and analyzed the expression data using the limma package for the R programming environment [68]. The total gene signal from the GeneView result files, which summarized the measurements of all probes for each gene on an array, was used in the analysis. Only those genes which were detected in at least 90% of the samples in either HCV+ or HCV- groups were kept in the further analysis. The detection of a gene was based on the "gIsGeneDetected" column. The expression data was normalized across arrays using median centered approach. To damp down the variability for low-intensity genes, an offset of 5 was used to add a constant to the signals before log-transforming. To avoid missing values after log-transformation, any signal which was still less than 0.5 was reset to be equal to 0.5. A batch factor was included in the linear model to remove potential batch effects. Differential expression was assessed by using moderated t-statistics.

#### mRNA microarray analysis

The mRNA gene expression profiling was carried out using the Agilent Human 1A (V2) 22 K oligonucleotide expression arrays. Each microarray experiment was done with four technical replicates by reversing dye hybridization for experimental and reference samples. The same reference sample was used for all hybridizations. Slides were scanned with an Agilent DNA microarray scanner, and image data were processed using Agilent Feature Extractor Software, which also performed error modeling. All data were subsequently uploaded into Rosetta Resolver (Rosetta Biosoftware, Seattle, WA) for data analysis. The Resolver system performs a squeeze operation that creates ratio profiles by combining replicates while applying error weighting. The error weighting consists of adjusting for additive and multiplicative noise. The Resolver system then combines ratio profiles to create ratio experiments using an error-weighted average as described in Roland Stoughton and Hongyue Dai, Statistical Combining of Cell Expression Profiles (US Patent #6,351,712, February 26, 2002). For each microarray experiment, the calculation of mean ratios between expression levels of each gene in the analyzed sample pair, standard deviations, and *P *values was performed using Resolver. All microarray data have been deposited in the GEO database with the SuperSeries accession number GSE15387. All data described in this report are also publicly available at http://viromics.washington.edu, in accordance with proposed MIAME standards.

### Discovery of miRNA-mRNA regulatory modules

#### miRNA-mRNA correlation network

In the miRNA-mRNA correlation network, there are two sets of vertices (nodes). One set of vertices are miRNAs, and another set are mRNAs. An unweighted and undirected edge (connection) is put between a pair of miRNA-mRNA genes if those two are co-expressed across different conditions (liver biopsy samples) with a correlation better than a specified threshold. We first calculated a miRNA-mRNA correlation matrix, where the rows were mRNAs, and the columns were miRNAs. All miRNA-mRNA pair correlations were calculated using the standard Pearson method. Correlation coefficients between gene pairs involving fewer than 80% (24/30) of data points (samples) were discarded due to missing values. Based on a selected threshold, the calculated correlation matrix was converted into a binary matrix of the same size. An entry in the binary matrix was 1 if the corresponding correlation was better than or equal to the threshold, otherwise the entry was 0. Only those vertices, both miRNAs and mRNAs, having at least one connection to other vertices were kept in the final network.

#### miRNA-mRNA regulatory network

To investigate if miRNAs directly regulated the mRNAs with which they were highly correlated as defined above, we superimposed miRNA target predictions onto the miRNA-mRNA correlation network. We only kept those entries where the mRNAs (rows) were the predicted targets of the corresponding mRNAs (columns) in the miRNA-mRNA correlation network. The resulting network was defined as a miRNA-mRNA regulatory network, essentially a bipartite graph. Similarly, there were two disjoint sets of vertices in this network, miRNAs and mRNAs. A direct connection was placed from a miRNA to an mRNA when: a) the expression level of the miRNA was highly correlated to that of the mRNA, and b) the mRNA was predicted to be the target of the miRNA.

#### Search for miRNA-mRNA regulatory modules

To uncover if multiple miRNAs co-ordinately regulate groups of mRNAs, we searched for miRNA-mRNA regulatory modules. Here, a miRNA-mRNA regulatory module is defined as a set of miRNAs and a set of mRNAs, in which each mRNA is both a target of, and highly correlated to all of the miRNAs in the module. Since the constructed miRNA-mRNA regulatory network is a bipartite graph, a module in the network corresponds to a biclique in the bipartite graph. A biclique, or a complete bipartite graph, is a special kind of bipartite graph, where every vertex in the first set (miRNAs) is connected to every vertex in the second set (mRNAs) [69]. We enumerated all maximal bicliques in the miRNA-mRNA regulatory network using [70]. Each identified biclique was considered as a candidate miRNA-mRNA regulatory module.

#### miRNA target prediction

We predicted miRNA targets based on the seed matches. The seed region of a miRNA is defined as positions 2–7 of a mature miRNA numbered from its 5' end [71]. When the 3' UTR sequence of an mRNA contains a perfect match to the seed of a miRNA, the mRNA is considered as a predicted target of the miRNA. Those seed matches are further classified into four classes in order: matches to positions 2–8 with 'A' in position 1 (8-mer), 2–8 (7-mer), 2–7 with 'A' in position 1 (2–7.A1), and 2–7 (6-mer) [71]. The 3' UTR sequences and the corresponding mapping between different gene identifiers were downloaded from Ensembl (Ensembl 52) using BioMart [72]. For comparisons, two publicly available target predictions, miRBase (version 5) [73] and TargetScan (release 4.2) [71], were obtained from their respective Web sites.

### Post-processing and statistical analysis

#### Generation of random datasets

To generate a random miRNA-mRNA expression dataset, we randomly chose, with replacement, the same number of miRNA microarray data from the original microRNA dataset to generate a 'random' miRNA microarray dataset. Similarly, we generated a random mRNA microarray dataset by randomly choosing the same number of mRNA microarray data from the original mRNA microarray dataset. Since the underling samples in the random miRNA microarray dataset do not correspond to the samples in the random mRNA microarray dataset, high miRNA-mRNA correlations obtained from this random miRNA-mRNA dataset are a result of chance and therefore are considered as false.

To generate a random set of miRNA target predictions, we first converted the real target predictions into a binary miRNA-target matrix. The rows are mRNAs and the columns are miRNAs. If mRNA *i *is a predicted target of miRNA *j *the corresponding entry *ij *is set to *1*, otherwise it is set to *0*. We then randomly permuted the rows of the miRNA-target matrix. This ensures that we keep the same distributions for both the numbers of target mRNAs per miRNA, and the number of miRNAs per mRNA. This also ensures that multiple miRNAs for the same mRNAs are correlated in the same way as in the real predictions.

#### Estimation of false detection rate

For a selected threshold for miRNA-mRNA correlations, we estimated the total number of miRNA-mRNA pairs with correlations better than or equal to the threshold found by chance using permutation-based tests. For each run, we generated a random miRNA-mRNA expression dataset as described above. We then calculated the corresponding miRNA-mRNA correlation matrix in the same way as described. The total number of miRNA-mRNA gene pairs with correlations satisfying the specified threshold was recorded as the number of false positives. We repeated the same process 100 times, and chose the median value of false positives as the estimated number of false positives associated with the threshold. Using the real miRNA-mRNA dataset we obtained the total number of miRNA-mRNA gene pairs with correlations satisfying the same threshold. The false detection rate associated with the threshold was defined as the ratio between the estimated number of false positives and the total numbers of selected miRNA-mRNA gene pairs from the real dataset. The false detection rates were calculated for a series of thresholds. The selection of a threshold was then based on a desired false detection rate.

#### Statistical significance of candidate regulatory modules

To evaluate if a candidate module can occur merely by chance, we assessed its statistical significances with two different metrics. For a candidate module, we first estimated the possibility of predicting equal or more numbers of targets by chance, given the same set of miRNAs. We generated a two-component random dataset as described above, a random miRNA-mRNA expression dataset and a random miRNA target prediction dataset. We then identified modules with this random dataset, using the same procedure and the same parameter values as we did on the real dataset. We recorded the number of targets predicted to be regulated by the same miRNAs in the candidate module. We repeated this same process 100 times. The percentage of times that equal or more targets were predicted to be regulated by the same miRNAs are considered as a *P *value for the candidate, denoted as P_Nt.

In parallel, we estimated the statistical significance of the same candidate module using a scoring based metric. A score was calculated for a pair of mRNA *i *and miRNA *j *with correlation *r*. The score for the *ij*-pair, *S*_*ij*_, consists of two components: a) the probability of being a target of the miRNA *j*, *P*_*t*_*j*_, b) the probability of having the same or better correlations than *r *between miRNAs and mRNAs, *P*_*cor*_*(r)*. The score *S*_*ij *_(Eq. 1) was defined as the product of these two, and then log transformed for the convenience of calculation:(1)

Essentially *S*_*ij *_represents the joint probability of being a target and having a high correlation, which are assumed to be independent. *S*_*ij *_is set to zero if mRNA *i *is not a predicted target of miRNA *j*, or the correlations between mRNA *i *and miRNA *j *is relatively too weak (defined as the *P *value of the correlation coefficient is greater than 0.01 here). The score for any set of *m *miRNAs and *t *mRNAs was calculated as the sum of scores of *mt *miRNA-mRNA gene pairs, with the assumption that every miRNA-mRNA pair is independent of others.

For every random dataset generated above, we estimated the corresponding distributions for both correlations and target predictions. For a candidate module with *m *miRNAs and *t *mRNAs, we calculated the score using the estimated distributions. For comparison, we enumerated all sets of *m *miRNAs and *t *mRNAs and found the one with the maximum score from the random dataset. The paired student t-test was performed to test if the candidate module had significantly better scores than the best scores obtained from random datasets. The *P *value from the one-side t-test was the scoring based *P *value for the candidate module, P_score.

#### Differential expression of targets in candidate modules

We fit a two-way ANOVA model (Eq. 2) to evaluate if overall the targets of a candidate module were differentially expressed between HCV+ and HCV- samples. Let *y*_*ijk *_be the normalized expression levels for the *i*_*th *_gene, *j*_*th *_treatment and *k*_*th *_replicates of this gene, we model gene effect *ψ*_*i*_, treatment effect *τ*_*j *_as two factors having *i *and *j *levels, *i *= 1, 2,..., *I*, *j *= 1, 2,..., *J *where *I *represents the number of genes in the candidate module and *J *represents the number of conditions to compare (HCV+ vs. HCV- here):(2)

To choose an appropriate cutoff p-value for the treatment effect *τ*, we randomly selected the same number of genes as in the candidate module from the mRNA microarray dataset and fit the ANOVA model. We repeated the process 1,000 times. The percentage of times obtaining a *P *value less than that of the candidate module was considered as the adjusted *P *value, assuming the majority of genes represented on the array were not differentially expressed between HCV+ and HCV- samples.

### Functional analysis of identified regulatory modules

#### Protein interaction network

The protein-protein interaction network data was downloaded from BioGRID (release 2.0.46). The NCBI Entrez GeneID was used to catalog interactions and redundant interactions were removed. The final network had 7,820 proteins and 24,828 interactions.

#### Enrichment analysis of functional groups

For the enrichment analysis of GO terms and KEGG pathways, we created a customized annotation package for all human Entrez Genes using AnnotationDbi, a Bioconductor package in R [74]. Enrichment was assessed using a one-sided hypergeometric test, using the GOstats [75]. We also used Ingenuity Pathways Analysis software (Ingenuity Systems, CA) for pathway analysis. IPA analyzes gene sets in the context of manually curated pathways and networks. Fisher's exact test was used to select significant pathways.

## Abbreviations

HCV: hepatitis c virus; miRNA: microRNA; GO: Gene Ontology. IPA: Ingenuity Pathways Analysis.

## Authors' contributions

XP, YL, SP and MGK designed research. YL, KAW, SLL, LDA and ERR performed experiment. XP made prediction. XP, YL and SP analyzed data. XP, YL, SP and MGK wrote manuscript. MGK supervised study. All authors read and approved the final manuscript.

## Supplementary Material

Additional file 1 **Supplement tables and figures**. This file contains all supplementary tables, figures and related descriptions. Click here for file

Additional file 2 **Complete lists of genes in identified modules**. This file includes tab-delimited text files, one for each identified module, listing all miRNAs and mRNAs and the correlation coefficients between them. It also has the details on target prediction, including the class of seed match and comparison with predictions from miRBase and TargetScan. Click here for file
