# Supplementary material for: Computational identification of hepatitis C virus associated microRNA-mRNA regulatory modules in human livers
Source: BMC Genomics. 2009 Aug 11;10:373. doi: 10.1186/1471-2164-10-373 (PMC2907698; doi:10.1186/1471-2164-10-373)
Supplement: Additional file 1 — Supplement tables and figures. This file contains all supplementary tables, figures and related descriptions. [file 1471-2164-10-373-S1.doc]

# Additional data file 1

## Figure S1 - Degree distributions of constructed networks

A, miRNA-mRNA correlation network. On the left the histogram shows the distribution of the number of miRNAs highly correlated to each mRNA. The right one shows the distribution of the number of mRNAs highly correlated to each miRNA. **B.** miRNA-mRNA regulatory network, similar as in (**A**). For each histogram, the numbers on the upper right corner show the summary statistics: maximum, minimum, average and median of the corresponding distributions.

## Figure S2 - Summary statistics of maximal bicliques identified in miRNA-mRNA regulatory network

**A,** Size distributions of identified bicliques. The x-axis shows the number of target mRNAs in a biclique, and the y-axis for the number of miRNAs in the same biclique. **B.** Histogram of the number of target mRNAs, and **C.** Histogram of the number of regulatory miRNAs in each biclique.

## Figure S3 - Summary statistics of selected bicliques based on the minimum number of targets

**A.** Median expression ratios of HCV+ vs. HCV- samples of miRNAs in selected bicliques. The x-axis shows the minimum number of targets for a biclique to be selected. **B** Mean expression ratios, similarly as in (**A**). **C.** Percentage of total targets covered by selected bicliques, as compared to those without the requirement for the minimum number of targets per biclique. **D.** Percentage of total miRNA gene families covered by all selected bicliques, similarly as in (**C**).

## Table S1 - Summary of miRNAs selected for module identification

HCV+: HCV positive samples. HCV-: HCV negative samples. Predicted modules: ‘X’ indicates that at least one miRNA-mRNA module included the corresponding miRNA.
